# Single- and Dual-Species Biofilm Formation by Shiga Toxin-Producing *Escherichia coli* and *Salmonella*, and Their Susceptibility to an Engineered Peptide WK2

**DOI:** 10.3390/microorganisms9122510

**Published:** 2021-12-03

**Authors:** Zhi Ma, Xia Tang, Kim Stanford, Xiaolong Chen, Tim A. McAllister, Yan D. Niu

**Affiliations:** 1 College of Biotechnology and Bioengineering, Zhejiang University of Technology, Hangzhou 310014, China; mazhi@zjut.edu.cn (Z.M.); 18268823075@163.com (X.T.); 2 Agriculture and Agri-Food Canada, Lethbridge Research and Development Centre, Lethbridge, AB T1J 4B1, Canada; tim.mcallister@AGR.GC.CA; 3 Department of Biological Science, University of Lethbridge, Lethbridge, AB T1J 4V6, Canada; kim.stanford@uleth.ca; 4 Department of Ecosystem and Public Health, Faculty of Veterinary Medicine, University of Calgary, Calgary, AB T2N 4Z6, Canada

**Keywords:** mixed-species biofilm, Shiga toxin-producing *Escherichia coli* (STEC), *Salmonella*, antimicrobial peptide

## Abstract

Shiga toxin-producing *Escherichia coli* (STEC) and *Salmonella enterica* are important foodborne pathogens capable of forming both single- and multi-species biofilms. In this study, the mono- and dual-species biofilms were formed by STEC O113:H21 and *Salmonella enterica* serovar Choleraesuis 10708 on stainless steel in the presence of beef juice over 5 d at 22 °C. The dual-species biofilm mass was substantially (*p* < 0.05) greater than that produced by STEC O113:H21 or *S.* Choleraesuis 10708 alone. However, numbers (CFU/mL) of *S*. Choleraesuis 10708 or STEC O113:H21 cells in the dual-species biofilm were (*p* < 0.05) lower than their respective counts in single-species biofilms. In multi-species biofilms, the sensitivity of *S*. Choleraesuis 10708 to the antimicrobial peptide WK2 was reduced, but it was increased for STEC O113:H21. Visualization of the temporal and spatial development of dual-species biofilms using florescent protein labeling confirmed that WK2 reduced cell numbers within biofilms. Collectively, our results highlight the potential risk of cross-contamination by multi-species biofilms to food safety and suggest that WK2 may be developed as a novel antimicrobial or sanitizer for the control of biofilms on stainless steel.

## 1. Introduction

Shiga toxin-producing *Escherichia coli* (STEC) strains are important foodborne pathogens, with beef products and fresh produce being most frequently adulterated. STEC can cause bloody diarrhea and other severe diseases, such as hemolytic uremic syndrome (HUS), the leading cause of acute renal failure in children [1]. STEC O157:H7 is the most commonly identified serotype associated with foodborne outbreaks and clinical disease, but there has been a growing incidence of non-O157 STEC infections in America since 2013 [2]. The severity of illnesses caused by non-O157 STEC such as O26, O45, O91, O103, O111, O113, and O145 may equal or even exceed that associated with STEC O157:H7. Meanwhile, *Salmonella enterica* infections have been reported as the second leading cause of bacterial foodborne illness in the United States, responsible for approximately 11% of all infections caused by foodborne pathogens. Over 95% of human salmonellosis cases have been associated with the consumption of contaminated foods [3]. It has been estimated that human infections by *S*. *enterica* are responsible for approximately 1.0 million clinical cases each year in the United States, resulting in over 19,000 hospitalizations and 378 deaths [3].

Biofilm formation is one of the major strategies that supports bacterial survival [4]. In nature, bacteria are able to form single-species biofilms or, more frequently, coexist in multi-species communities and form mixed biofilms on various food products and contact surfaces. It has been shown that multiple bacterial species, including *E. coli*, *Salmonella*, *Pseudomonas*, *Staphylococcus*, and *Listeria*, can coexist and form multi-species biofilms in food processing plants [5,6]. Numerous investigations have shown that STEC and *Salmonella* can be simultaneously isolated from cattle hides, feces, and carcasses in meat processing plants of varying capacity [7,8]. Furthermore, fresh produce has been increasingly implicated in foodborne outbreaks caused by STEC and *Salmonella* [9], and multi-species biofilms of Gram-positive and Gram-negative bacteria were also found on the leaves of different fresh vegetables, such as spinach, parsley, leeks, and lettuce [10]. These findings indicate that the coexistence of these two important and frequently isolated foodborne pathogens in food processing environments poses a potential food safety concern.

Previous reports have revealed that multiple-species coexistence could significantly affect bacterial biofilm development and its structure. Uhlich et al. [11] showed that the ability of *E. coli* O157:H7 to form biofilms was enhanced if it formed a mixed biofilm with a companion *E. coli* O:H4 strain and that its sensitivity to treatment with 5% hydrogen peroxide was reduced. However, Chen et al. [12] found that *E. coli* O157:H7 USDA 5 and *Salmonella* 45788 isolates were antagonistic within dual-species biofilms, and the resistance of both pathogens in dual-species biofilms to levulinic acid plus SDS was decreased as compared to single-species biofilms.

The removal and inactivation of foodborne pathogens in single- and mixed-species biofilms are critical for improving hygiene, controlling contamination, and enhancing food safety [13]. In recent years, antimicrobial peptides (AMPs) have been shown to effectively inhibit biofilm formation by *E. coli*, *Salmonella*, and *S. aureus* [14]. However, there are few reports concerning the activity of AMPs against mixed-species biofilms formed by foodborne pathogens, even though mixed-species biofilms are more frequent in meat processing plants than single-species biofilms [15]. In our previous study, the engineered peptide WK2 with sequence (WK)_2_CTKSGC(KW)_2_ displayed potent antimicrobial and antibiofilm activities against *Salmonella* [16]. This finding motivated us to explore the activities of this peptide against mixed-species biofilms formed by STEC and *Salmonella*. Therefore, the objective of this study was to gain insight into the impact of engineered peptide WK2 on the competition, development, and stability of dual-species biofilms formed by STEC O113:H21 and *Salmonella enterica* subspecies *enterica* serovar Choleraesuis ATCC 10708.

## 2. Materials and Methods

### 2.1. Bacterial Strains and Cultivation

STEC EC20020170 O113:H21 and *S.* Choleraesuis ATCC 10708 were used in this study, as these two strains were previously identified as strong biofilm producers on stainless coupons in beef juice (Figure S1; [17]). Cultures were stored at −80 °C in Luria–Bertani broth (LB; Sigma-Aldrich, Oakville, ON, Canada) containing 25% glycerol (Sigma-Aldrich). Cultures were thawed and streaked on LB agar plates (Sigma-Aldrich). After 18–20 h incubation, a well-isolated colony was selected and inoculated into 10 mL of LB and incubated at 37 °C at 180 rpm for 18 h.

### 2.2. Food Contact Surface Materials and Preparation of Beef Juice

The test surface was 304 stainless steel coupons, finish No. 4 (2.54 cm × 3.81 cm × 0.081 cm, Biosurface, Bozeman, MT, USA). The coupons were prepared by soaking in 10% bleach for 5 min, thoroughly rinsing with deionized water, air-drying, and autoclaving. Beef juice was filter-sterilized as described previously [17], and the concentration of protein was measured by a biuret reagent kit (Sigma-Aldrich) and adjusted to 5 mg/mL using sterile water.

### 2.3. Preparation of Single- and Mixed-Species Cultures

Individual bacterial strains were grown in LB at 37 °C for 18–20 h. The single-species cultures were prepared using a 1:100 dilution of the overnight culture (ca. 9.0 log_10_ CFU/mL) in sterile beef juice. To prepare the mixed-species culture, an equal volume of the 1:100 diluted overnight culture of each strain at the same bacterial population density (7.0 log_10_ CFU/mL) was combined. Cell numbers of overnight culture were confirmed by dilution series plating on MacConkey agar (Becton Dickinson, Sparks, MD, USA).

### 2.4. Single- and Mixed-Species Biofilm Formation on Stainless Steel Surface in Beef Juice

Single- and mixed-species cultures (20 mL, ~7.0 log_10_ CFU/mL) and a bacteria-free control (20 mL, beef juice) were introduced into 50 mL Falcon tubes containing a sterile stainless steel coupon. The tubes were statically incubated at 22 °C for 24, 72, or 120 h. After each incubation period, the stainless steel coupons were removed from the tubes using sterilized forceps and rinsed with 25 mL of sterile Milli-Q water thrice to remove loosely attached bacteria. Subsequently, coupons with adherent biofilms were fixed with 25 mL of absolute methanol (Sigma-Aldrich, St. Louis, MO, USA) for 15 min. The fixed coupons were then air-dried for 2 min and stained with 0.5% (w/v) crystal violet solution (CV, Sigma-Aldrich, St. Louis, MO, USA) on a rocker platform at 40 rpm for 15 min. After staining, coupons were washed with 25 mL of sterile Milli-Q water three times to remove excess CV solution, and air-dried at room temperature for 5 min. Subsequently, coupons were immersed in 25 mL of 33% glacial acetic acid (Sigma-Aldrich, St. Louis, MO, USA) and allowed to stand at room temperature for 15 min. The dissolved dye was measured at 590 nm using a spectrophotometer (BioTek, Winooski, VT, USA). Two technical replicates for each sample were conducted in each independent assay. The results were expressed as an average of the data from three independent assays.

### 2.5. Enumeration of the Planktonic and Attached Cells

To count the planktonic cells after each period, 1 mL of the single- and mixed-species cultures was removed from the Falcon tubes, serially diluted with 10 mM sterile phosphate buffered saline (PBS, pH 7.4), and plated on MacConkey agar, with STEC O113:H21 and *Salmonella* forming red and white colonies, respectively. The plates were incubated at 37 °C for 18 h. To enumerate the biofilm cells, coupons were rinsed three times with sterile Milli Q water as described above, immersed in 25 mL of sterile PBS (pH 7.4), and sonicated at 20 kHz for 10 min (M3800, Branson Ultrasonics, Brookfield, CT, USA). After sonication, the Falcon tubes containing coupons were vortexed and 1 mL suspension of the bacteria was pipetted, serially diluted (10^2^ and 10^6^-fold), and plated on MacConkey agar. The colonies were counted after incubation for 18 h at 37 °C.

### 2.6. Peptide Synthesis

WK2 was synthesized and purified by GL Biochem Corporation (Shanghai, China) through solid-phase methods using N-9-fluorenylmethyloxycarbonyl (Fmoc) chemistry [14]. The molecular weight (1854.28) was confirmed through matrix-assisted laser desorption/ionization time-of-flight mass spectroscopy. Circular dichroism indicated that WK2 assembled into a rigid β-hairpin or β-sheet structure in a simulated membrane environment (trifluoroethanol). Peptide purity was more than 95% according to analytical reverse-phase high-performance liquid chromatography. The peptide was dissolved in sterile deionized water and stored at −20 °C.

### 2.7. Peptide Testing

Minimal inhibitory concentrations (MICs) of WK2 and ε-polylysine (ThermoFisher, MA, USA) against STEC O113:H21 and *S.* Choleraesuis ATCC 10708 were determined using the broth microdilution method. Briefly, peptides were diluted in 0.2% bovine serum albumin (BSA) containing 0.01% acetic acid, and 50 µL of bacteria (5.0 log_10_ CFU/mL) in LB was added to 50 µL of serially diluted aliquots of the peptide in sterile 96-well plates. The microtiter plates were then incubated at 37 °C overnight. MICs were determined as the minimal concentration at which no visible bacterial growth was observed, and all experiments were conducted in triplicate.

### 2.8. Sensitivity of STEC O113:H21 and S. Choleraesuis ATCC 10708 in Single- and Mixed-Species Biofilms to WK2

After the single- and mixed-species biofilms were formed on coupons over 120 h of incubation, they were rinsed and air-dried as described above, and then immersed in WK2 (16 µg/mL) or ε-polylysine (Sigma-Aldrich, 16 µg/mL) solutions for 5, 30, and 60 min. After treatment, residual biofilms were rinsed three times, sonicated, serially diluted, and 100 µL of samples were plated in duplicate on MacConkey agar, as described above. The colonies were counted after incubation at 37 °C for 18 h. The minimum limit of detection by the direct plating method was 0.7 log CFU/mL.

### 2.9. Confocal Laser Scanning Microscopy (CLSM)

To enable visualization of STEC O113:H21 and *S.* Choleraesuis ATCC 10708 in dual-species biofilms using CLSM, strains were transformed with plasmids encoding two different fluorescent proteins. The plasmids pGFPuv (green fluorescence) and pDsRed-Express2 (red florescence; Clontech Laboratories, Mountain View, CA, USA) were transformed into STEC O113:H21 and *S.* Choleraesuis ATCC 10708, respectively. Both plasmids possessed an ampicillin marker, the resulting STEC O113:H21 strain produced green fluorescence under UV light, and the resulting *Salmonella* 10708 strain produced red fluorescence. The two plasmids remained stable in the respective strains after six consecutive days of sequential propagation in LB medium with ampicillin.

Overnight cultures of FP-labeled O113:H21 and 10708 were diluted in beef juice (containing 100 mg/mL of ampicillin) and added into each well of a 6-well plate (2 mL, 1 × 10^5^ CFU/mL) containing a 25 mm round glass coverslip (Fisher Scientific, Pittsburgh, PA). The bacterial cultures were statically incubated at 22 °C for 120 h. Following incubation, the coverslips were carefully removed from the medium using sterile forceps, gently rinsed three times with sterile PBS, treated with WK2 and ε-polylysine for 5, 30, and 60 min, and washed again with PBS. The samples were visualized under a Confocal Laser Scanning Microscope (Carl Zeiss Microscopy, Jena, Germany) using a Plan-Apochromat 63 x oil-immersion, numerical aperture 1.4 objective lens (Carl Zeiss) coupled to a 488 nm argon laser and a 555 nm diode-pumped solid-state laser. STEC O113:H21 (green) was visualized at a bandwidth of 490–550 nm (green) and *S.* Choleraesuis 10708 at 560–700 nm (red). Z-stack scanning was performed to visualize the spatial distribution of the two strains within biofilms.

### 2.10. Statistical Analysis

All the experiments were conducted three times independently. Data were analyzed using PROC MIXED and least-squares differentiated means with SAS (Statistical Analysis Systems Institute, Cary, NC, USA). Significant differences are presented at a 95% confidence level (*p* ≤ 0.05).

## 3. Results

### 3.1. Single- and Mixed-Species Biofilm Mass Measured by CV Staining

**Figure 1:**
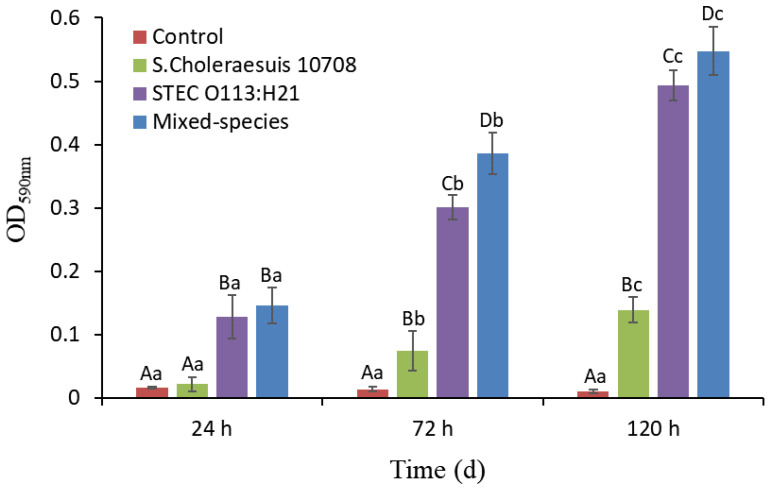
Single- and mixed-species biofilm formation by STEC O113:H21 and *S.* Choleraesuis ATCC 10708 on stainless steel surface in beef juice at 22 °C for 5 days. Biofilm-forming ability was quantified by crystal violet staining and absorbance measurement at 590 nm. Uppercase and lowercase letters represent that mean OD_590_ values differ (*p* < 0.05) among species of biofilm within each time (A–D) and over time within each species of biofilm (a–c). Error bar represents the 95% confidence interval for the mean. OD: optical density.

Despite the bacterial species, the biofilm mass on the stainless steel coupon gradually increased (*p* < 0.05) over the period of 120 h incubation at 22 °C (Figure 1). Moreover, the biofilm mass produced by mixed species was greater (OD_590_ = 0.12, *p* < 0.05) than that produced by single species after 24 h (*S*. Choleraesuis 10708). After 72 and 120 h, the biofilm mass produced by mixed species was greater (OD_590_ = 0.37 − 0.53 for *S*. Choleraesuis, OD_590_ = 0.06 − 0.08 for STEC; *p* < 0.05) than that produced by both single species tested in the study. In addition, STEC O113:H21 formed greater biofilm mass (OD_590_ = 0.10 − 0.35, *p* < 0.05) than *S*. Choleraesuis at each sampling time.

### 3.2. Enumeration of the Single- and Mixed-Species Planktonic and Attached Bacterial Cells

**Figure 2:**
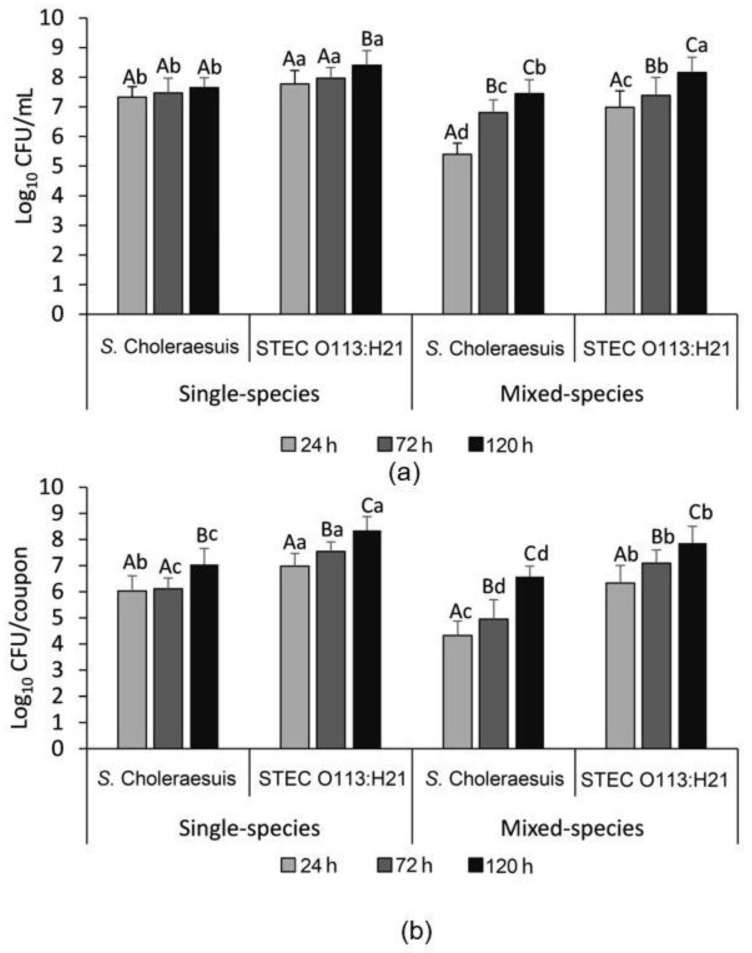
Bacterial counts of STEC O113:H21 and *S.* Choleraesuis 10708 in their respective mono- and mixed-species planktonic cultures (**a**) and biofilms (**b**). Within each time, means with different lowercase letters differ (*p* < 0.05). Within each species, means with different uppercase letters differ (*p* < 0.05). Error bar represents the 95% confidence interval for the mean.

As shown in Figure 2a, *S.* Choleraesuis 10708 reached a stationary phase within 24 h in the mono-species planktonic culture at 7.5 log_10_ CFU/mL. In contrast, the growth of *S.* Choleraesuis 10708 only reached 5.4 log_10_ CFU/mL in the mixed-species planktonic culture after 24 h, but increased (*p* < 0.05) to 7.4 log CFU/mL after 120 h of incubation, a density similar (*p* > 0.05) to when it was grown individually. For STEC O113:H21, populations of planktonic cells reached 7.8 log_10_ CFU/mL in the mono-species planktonic culture after 24 h of incubation, and increased (*p* < 0.05) to 8.4 log CFU/mL by 120 h. STEC O113:H21 grew to 7.0 log_10_ CFU/mL in the mixed-species planktonic culture within 24 h, but growth was lower as a single-species culture. However, after 120 h, it reached 8.2 log_10_ CFU/mL, a density similar (*p* > 0.05) to its growth as a single species.

In single-species biofilms, cell numbers of *S.* Choleraesuis 10708 and STEC O113:H21 reached 6.0 and 7.0 log CFU/mL after 24 h, and increased (*p* < 0.05) to 7.3 and 8.4 log CFU/mL after 120 h of incubation, respectively (Figure 2b). However, in mixed-species biofilms, the biofilm cells of *S.* Choleraesuis 10708 and STEC O113:H21 were 4.3 log_10_ CFU/mL and 6.3 log_10_ CFU/mL at 24 h, and reached 6.6 log_10_ CFU/mL and 7.9 log_10_ CFU/mL after 120 h, respectively. This cell density was lower (*p* < 0.05) than their respective counts recovered from single-species biofilms. Overall, STEC O113:H21 formed a biofilm with more (*p* < 0.05) adherent cells than *S.* Choleraesuis 10708 in both single- and mixed-species conditions.

### 3.3. The Susceptibility of S. Choleraesuis 10708 and STEC O113:H21 in Single- and Mixed-Species Biofilms to WK2

The antimicrobial activity of WK2 was evaluated against a planktonic culture of *S.* Choleraesuis 10708 and STEC O113:H21, as well as against the biofilms formed by these strains. Peptide WK2 exhibited strong antibacterial activity against STEC O113:H21 and *S.* Choleraesuis 10708 planktonic cells, with an MIC of 4 and 16 μg/mL, respectively—an MIC concentration that was comparable to ε-polylysine (8 and 16 μg/mL, respectively).

**Figure 3:**
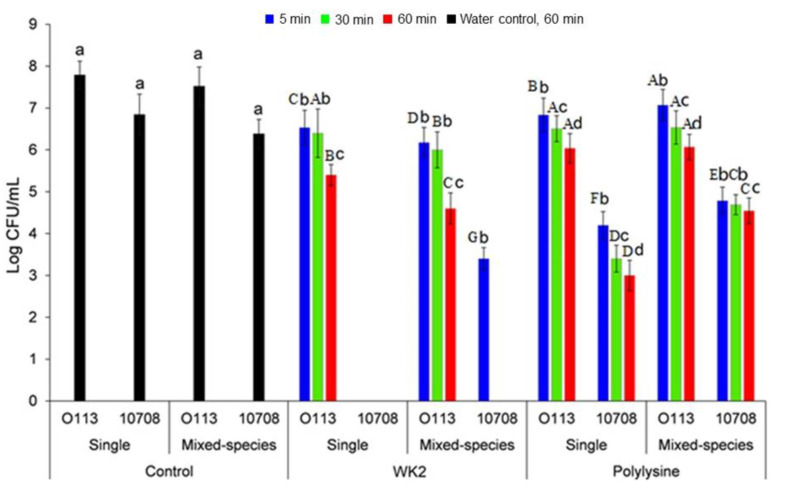
Bacterial counts of STEC O113:H21 and *S*. Choleraesuis 10708 in single- and mixed-species biofilms after treatment for 5, 30, and 60 min with WK2 (16 µg/mL) and polylysine (16 µg/mL). The samples treated by water for 60 min were set as controls. Within each time, means with different uppercase letters differ (*p* < 0.05). Within each species, means with different lowercase letters differ (*p* < 0.05).

The peptide WK2 reduced (*p* < 0.05) the number of STEC O113:H21 cells in the single-species biofilm by 1.2 log CFU/mL after 5 min, and 2.4 log CFU/mL after 60 min (Figure 3). Compared to STEC O113:H21, *S.* Choleraesuis 10708 cells in the single-species biofilm were more susceptible to WK2, which was completely eliminated after 5 min. Polylysine reduced the amount of STEC O113:H21 and *S.* Choleraesuis 10708 cells in single-species biofilms by 1.8 and 3.8 log CFU/mL after 60 min, respectively, reflecting its lower potency than WK2. In mixed-species biofilms, WK2 reduced (*p* < 0.05) STEC O113:H21 cells by 2.0 log CFU/mL within 60 min, suggesting that STEC O113:H21 cells in mixed-species biofilms were more sensitive to WK2 than those in single-species biofilms. By contrast, 3.4 log_10_ CFU/mL of *S.* Choleraesuis 10708 remained in the mixed-species biofilm after 5 min exposure to WK2, indicating that *S.* Choleraesuis 10708 was less sensitive to WK2 in a mixed biofilm. Differing from WK2, STEC O113:H21 in the mixed-species biofilm remained equally (*p* > 0.05) sensitive to polylysine as it did in the single-species biofilm, with 6.1 log_10_ CFU/mL being recovered after 60 min. Similarly, *S.* Choleraesuis 10708 cells in mixed-species biofilms appeared to be more resistant to polylysine than those in single species, with 4.5 log_10_ CFU/mL remaining on the coupon after 60 min.

### 3.4. Microscopic Examination of Single- and Mixed-Species Biofilms

**Figure 4:**
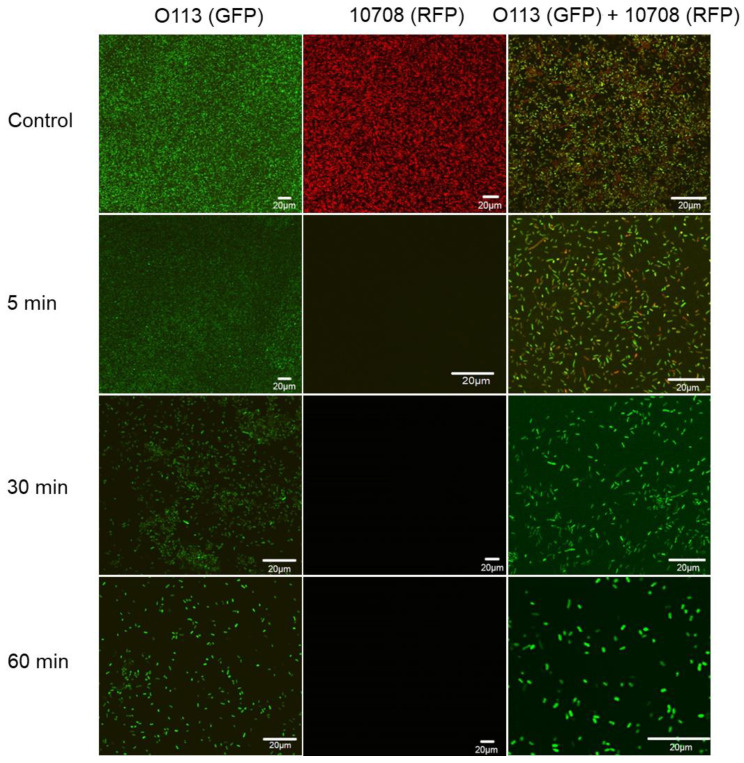
Representative photomicrographs of single- and mixed-species biofilms formed by GFP-labeled STEC O113:H21 and RFP-labeled *S.* Choleraesuis 10708 after treatment with WK2 for 0 (control), 5, 30, and 60 min. Scale bar = 20 µm.

Single- and mixed-species biofilms formed by GFP-labeled O113 and RFP-labeled 10708 on glass slides were examined using CLSM. As shown in Figure S2, the FP-labeled STEC O113:H21 and *S.* Choleraesuis 10708 cells in single- and mixed-species biofilms displayed green and red fluorescence, respectively. Cells were evenly distributed within multi-layered communities after 120 h of biofilm formation. However, after treatment with WK2 for 5, 30, and 60 min, the amount of pathogens in both single- and mixed-species biofilms dramatically decreased over time (Figure 4), and WK2 was more effective against *S.* Choleraesuis 10708 than STEC O113:H21, an observation that aligns with the impact of WK2 on the cell number (Figure 3).

## 4. Discussion

Biofilm formation is one of the strategies that bacteria use to withstand environmental stresses [18,19]. In nature, biofilms typically are composed of multiple species of bacteria, including foodborne pathogens, and are frequently formed in food processing environments [15,20]. A better understanding of the interaction among speciesand how to effectively remove mixed-species biofilms from abiotic surfaces in food processing facilities could help to reduce the risk of food contamination. In this study, static biofilms were formed on stainless steel and glass slides as this contact surface is frequently used in meat processing environments in areas such as countertops, sinks, equipment, and cutting boards [21,22].

The number of STEC O113:H21 cells within single-species biofilms formed on stainless steel was greater than that formed by *S.* Choleraesuis 10708 in beef juice. A slower planktonic growth rate for *S.* Choleraesuis 10708 than STEC O113:H21 was observed in beef juice, which may have contributed to the lower cell density as biofilms were formed. In addition, previous studies revealed that bacterial biofilm formation was strain-dependent, and the process was influenced by a variety of environmental factors, such as temperature, oxygen, nutrients, substratum, and incubation time [23,24]. In mixed-species biofilms, biofilm mass as measured by CV staining was slightly enhanced, but with reduced cell numbers of STEC O113:H21 and *S*. Choleraesuis. This was different from a previous report [12], in which both CV staining and biofilm cell enumeration showed that the biofilm-forming abilities of STEC O157:H7 USDA 5 and *S*. Choleraesuis 45788 in the mixed species were less profound than their counterparts in the single-species biofilm after incubation for 72 h at 21 °C in tryptic soy broth on a polystyrene surface. In this study, reduced cell numbers of STEC O113:H21 and *S*. Choleraesuis in the mixed species generated a similar growth outcome in their mixed-planktonic culture. The reduced biofilm cells in the mixed-species biofilm indicated that competitive interactions may have occurred between STEC O113:H21 and *S*. Choleraesuis [25]. The mechanism underlying the competition between STEC and *Salmonella* in the mixed-species biofilm remains poorly understood. However, Lories et al. [26] found that competition was associated with genes for biofilm formation, epithelial invasion, and antibiotic tolerance in the mixed-species biofilm of *S*. Typhimurium SL1344, ATCC14028, and *E. coli* K12. Furthermore, when recovering biofilm-forming cells from the mixed species, there appeared to be an inhibition zone surrounding *S*. Choleraesuis colonies (Figure S3), suggesting that diffusible molecules might be produced by *S*. Choleraesuis that could inhibit STEC O113:H21 growth under the co-culturing conditions. Further investigation is required to identify the secretion of molecules and their inhibitory effects.

In general, as compared to their parental strains, FP-labeled STEC O113:H21 and *S.* Choleraesuis performed similarly in the mixed-planktonic and biofilm phases, although their individual growth pattern and/or biofilm formation slightly changed (Figures S4–S6). Changes in growth pattern and biofilm formation due to the inclusion of FP were reported by others elsewhere. For example, FP-labeled STEC O157:H7 USDA 5 and *S*. Enteritidis 457–88 formed a weaker biofilm than their parental strains [12]. Expression of the introduced plasmids may pose a metabolic burden that lowers the growth rate of this bacterium within biofilms. However, in this study, FP enhanced the biofilm formation of *S.* Choleraesuis. Biofilm formation promotes plasmid stability and may enhance the host range of mobile genetic elements that are transferred [27]. On the other hand, plasmids are very well-suited to promote the evolution of social traits such as biofilm formation [27,28].

Bacteria may achieve high numbers on a contact surface as a result of high affinity with no or slow growth, or with low affinity but high growth rates post-attachment [29]. Our studies have focused on eliminating preformed biofilms on food processing contact surfaces by developing a novel disinfectant in the form of the peptide WK2. The results from bacterial enumeration and CLSM observations certified that WK2 dramatically obliterated the single- and mixed-species biofilms formed by STEC O113:H21 and *S. Choleraesuis* 10708. Compared to STEC O113:H21, WK2 exhibited higher activity against *S. Choleraesuis* 10708 within biofilms, although this activity was reduced within mixed-species biofilms. Previously, we have demonstrated that peptide WK2 binds to bacterial DNA, disrupts membrane integrity, and interferes with the AI-2-mediated quorum sensing system, reducing the viability of *Salmonella* within biofilms [16]. Interestingly, in mixed-species biofilms, STEC O113:H21 appeared to be more sensitive to WK2, whereas *S. Choleraesuis* 10708 cells exhibited increased resistance. In contrast, Wang et al. [30] observed that *E. coli* O157:H7 and *S.*Typhimurium in mixed-species biofilms were more resistant to quaternary ammonia compounds (QAC) than in single-species biofilms. This variable observation may have arisen as a result of several different factors. It is well-known that biofilm formation by STEC and *Salmonella* is strain-dependent [23,31]. In addition, the antagonistic effect observed between *S. Choleraesuis* 10708 and STEC O113:H21 in the mixed-species biofilm may have resulted in the enhanced sensitivity of STEC O113:H21 to WK2 compared to the single-species biofilms.

In conclusion, this study revealed that mixed-species biofilms formed by STEC O113:H21 and *S.* Choleraesuis 10708 were more robust than single-species biofilms. Interactions between STEC O113:H21 and *S.* Choleraesuis 10708 may occur in mixed-species planktonic cultures and biofilms, which change their sensitivity to antimicrobials such as WK2. The antimicrobial activity of WK2 against STEC and *Salmonella* in both planktonic and biofilm cells suggests that WK2 has potential as an alternative to chemical sanitizers for the effective decontamination of bacterial pathogens in biofilms within food processing facilities.

## Data Availability

Not applicable.

## Supplementary Materials

The following are available online at https://www.mdpi.com/article/10.3390/microorganisms9122510/s1: Figure S1: Biofilm formation of twelve Salmonella strains on stainless steel surface in the presence of beef juice at 22 °C (a) and their OD values (b); Figure S2: Representative photomicrographs of single- and dual-species biofilms formed by GFP-labeled STEC O113 and RFP-labeled S. Choleraesuis ATCC 10708; Figure S3: The growth of S. Choleraesuis 10708 and STEC O113:H21 cells in mixed-species biofilm on MacConkey agar; Figure S4: Comparison of planktonic growth rates of FP-labeled and parental STEC O113 and S. Choleraesuis ATCC 10708; Figure S5: Single- and mixed-species biofilm formation by FP-labeled and parental STEC O113:H21 and S. Choleraesuis 10708 on stainless steel surfaces incubated at 22 °C for 5 days; Figure S6: Cell enumeration of FP-labeled and parental STEC O113:H21 and S. Choleraesuis 10708 in their respective mono- and mixed-species planktonic cultures (a) and biofilms (b).
