# Supplementary material for: Single- and Dual-Species Biofilm Formation by Shiga Toxin-Producing Escherichia coli and Salmonella, and Their Susceptibility to an Engineered Peptide WK2
Source: Microorganisms. 2021 Dec 3;9(12):2510. doi: 10.3390/microorganisms9122510 (PMC8709450; doi:10.3390/microorganisms9122510)
Supplement: Supplementary file 1 [file microorganisms-09-02510-s001.zip › microorganisms-1463727-SI.pdf]

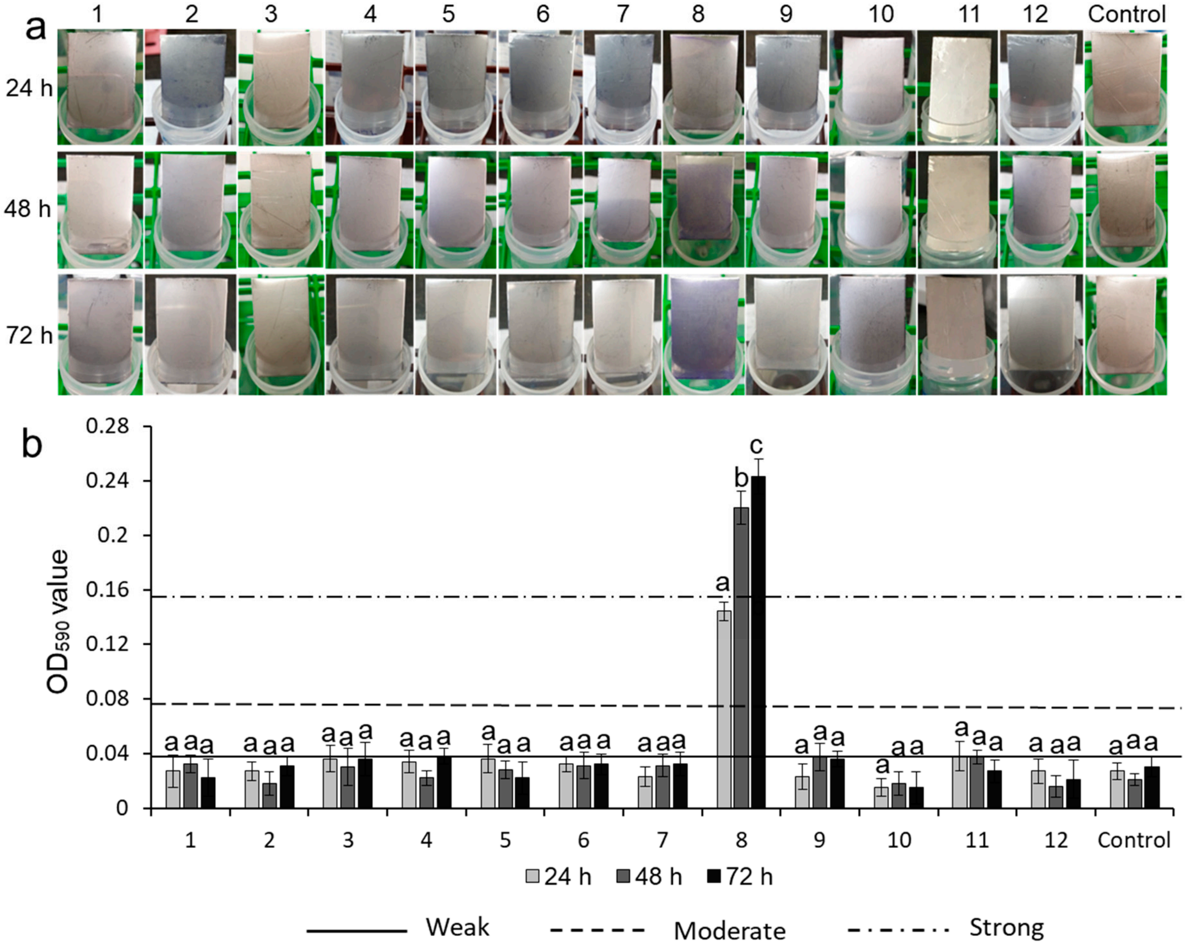

Figure S1. Biofilm formation of twelve *Salmonella* strains on stainless steel surface in beef juice at 22°C (**a**) and their OD values (**b**). The vertical axis represents the average of OD values, determined at 590 nm. Horizontal lines represent the cutoff values between weak, intermediate, and strong biofilm producers. The cutoff optical density value (ODc) of 0.039 is defined as three standard deviations above the mean OD of the negative controls. Strains were classified as OD ≤ ODc, no biofilm producer; ODc < OD ≤ 2 × ODc, weak biofilm producers; 2 × ODc < OD ≤ 4 × ODc, intermediate biofilm producers; and 4 × ODc < OD, strong biofilm producers. OD, optical density; STEC, Shiga toxin-producing *E. coli*. Means within a strain with different superscripts differ ($P < 0.05$). 1, 2, 3, 4, 5, 6, 7, 8, 9, 10, 11 and 12 represent *S.* Typhimurium 4,5:i:1,2, *S.* Enteritidis 9,12:g,m:-, *S.* Heidelberg 4:r:1,2, *S.* I:4,5,12:i:- 4.5:I:-, *S.* Saintpaul 4:e,h:1.2, *S.* Newport 6,8:e,h:1,2, *S.* Typhimurium ATCC 14028, *S.* Choleraesuis ATCC 10708, *S.* Dublin 1,9,12,[Vi]:g,p:-- ET Du2, *S.* Enteritidis 1,9,12:g,m:[1,7] ET En1, *S.* Heidelberg 1,4,[5],12:r:1,2 ET He1. Biotype 9i, *S.* Newport 6,8:e,h:1,2 ET Np8.

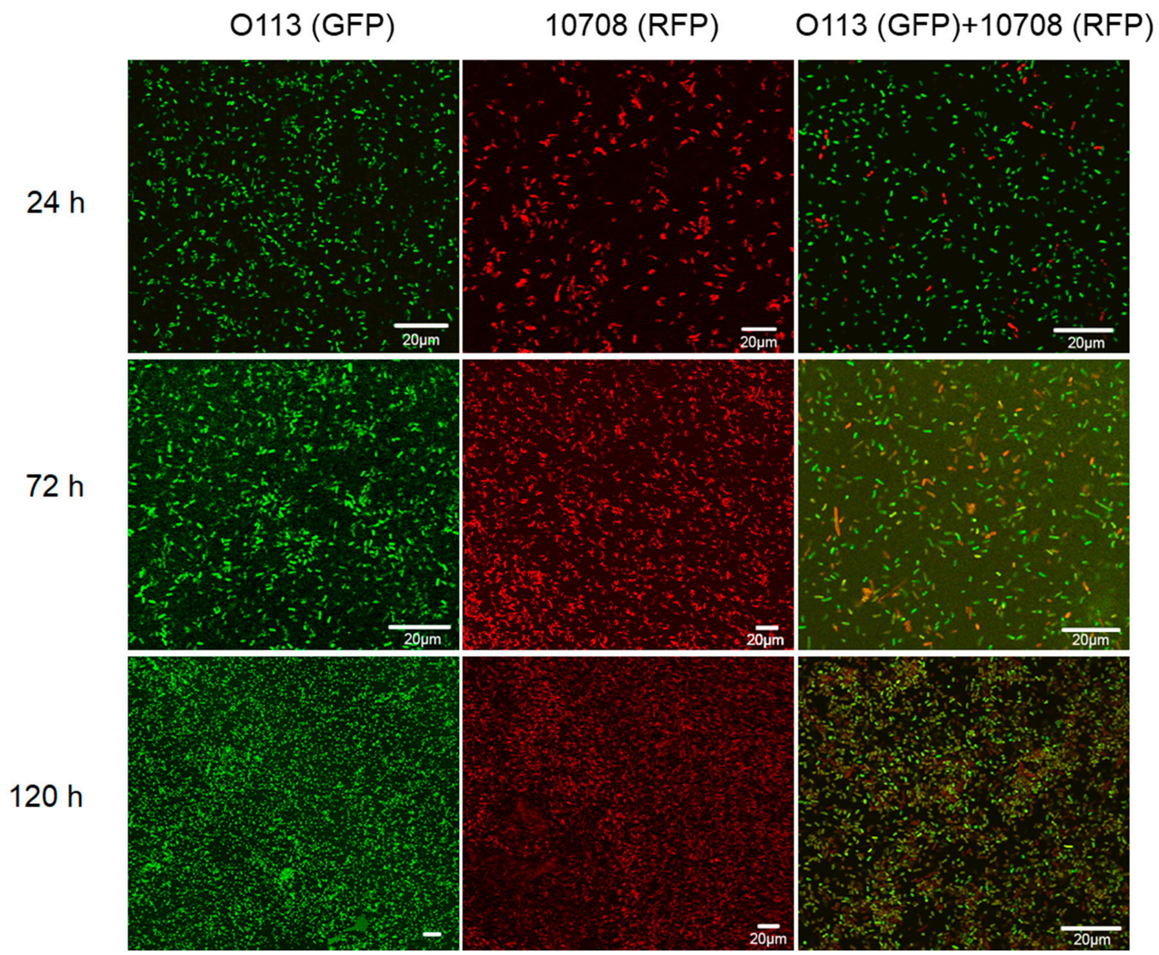

Figure S2. Representative photomicrographs by CLSM of single- and mixed-species biofilms formed by GFP-labeled STEC O113 and RFP-labeled *S*. Choleraesuis ATCC 10708. The biofilms were formed after 24, 72, and 120 h of incubation on stainless steel surface in beef juice at 22 °C. Scale bar = 20 μm.

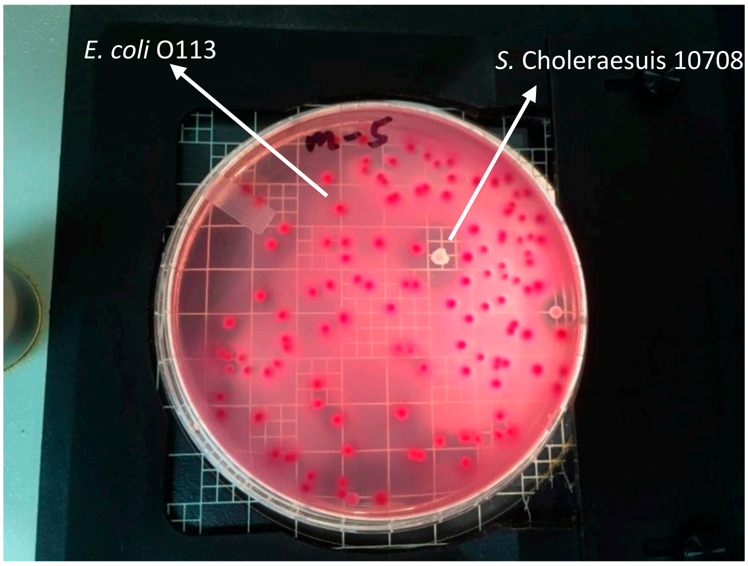

Figure S3. The growth of *S*. Choleraesuis 10708 and *E*. coli O113 cells in mixed-species biofilm on MacConkey agar. After incubation for 24 h, cells of these two pathogens in mixed-species biofilm were diluted and plated on MacConkey agar, with *E*. coli O113 and *S*. Choleraesuis 10708 forming red and white colonies, respectively.

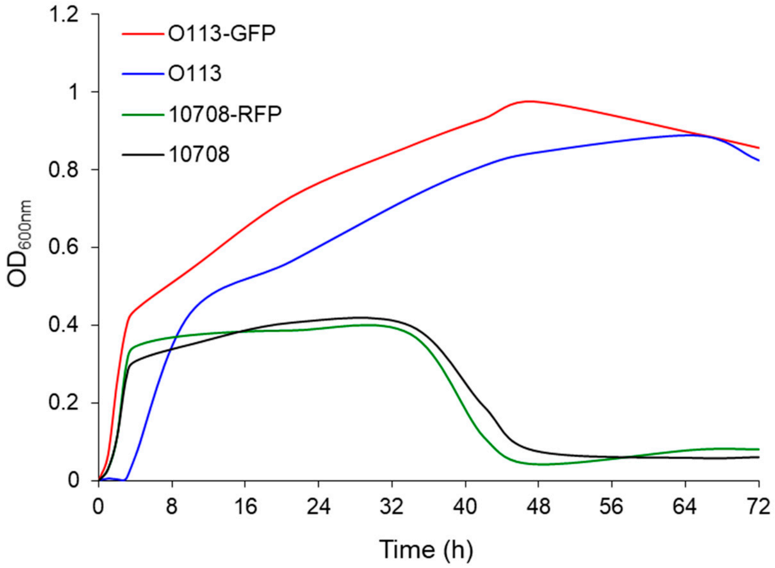

Figure S4. Comparison of planktonic growth rates of FP-labeled and parental STEC O113 and *S*. Choleraesuis ATCC 10708. Growth rates of FP-labeled and parental bacterial strains were determined by measuring absorbance at 600 nm per 30 min after 1:100 inoculation in LB without ampicillin. Point values are the means of at least three trials.

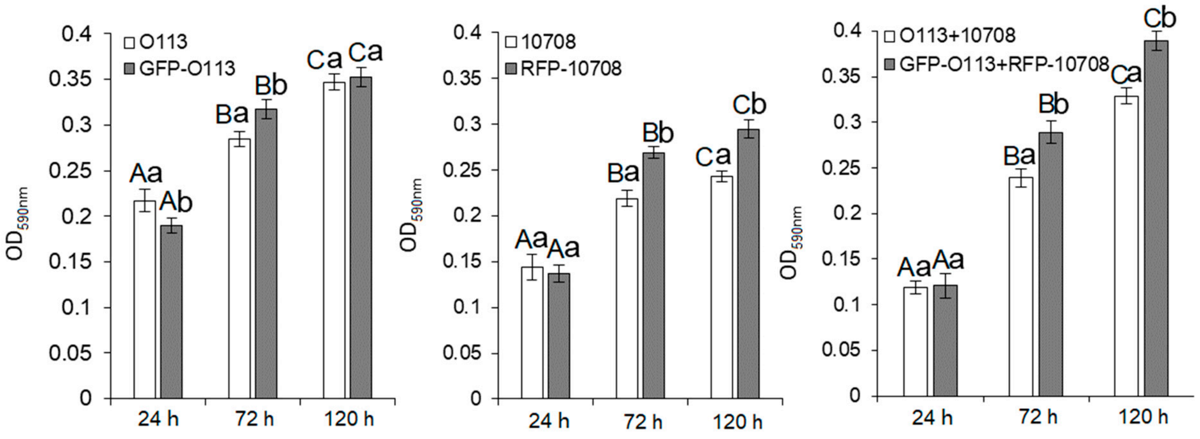

Figure S5. Single- and mixed-species biofilm formation by FP-labeled and parental *E. coli* O113 and *S.* Choleraesuis 10708 on stainless steel surfaces incubated at 22 °C for 120 h. Biofilm-forming ability was quantified by crystal violet staining with measurement at OD_{590nm}. The bars represent the mean values + standard deviations. Within each time, means with different lowercase letters differ ($P < 0.05$). Within each species, means with different uppercase letters differ ($P < 0.05$).

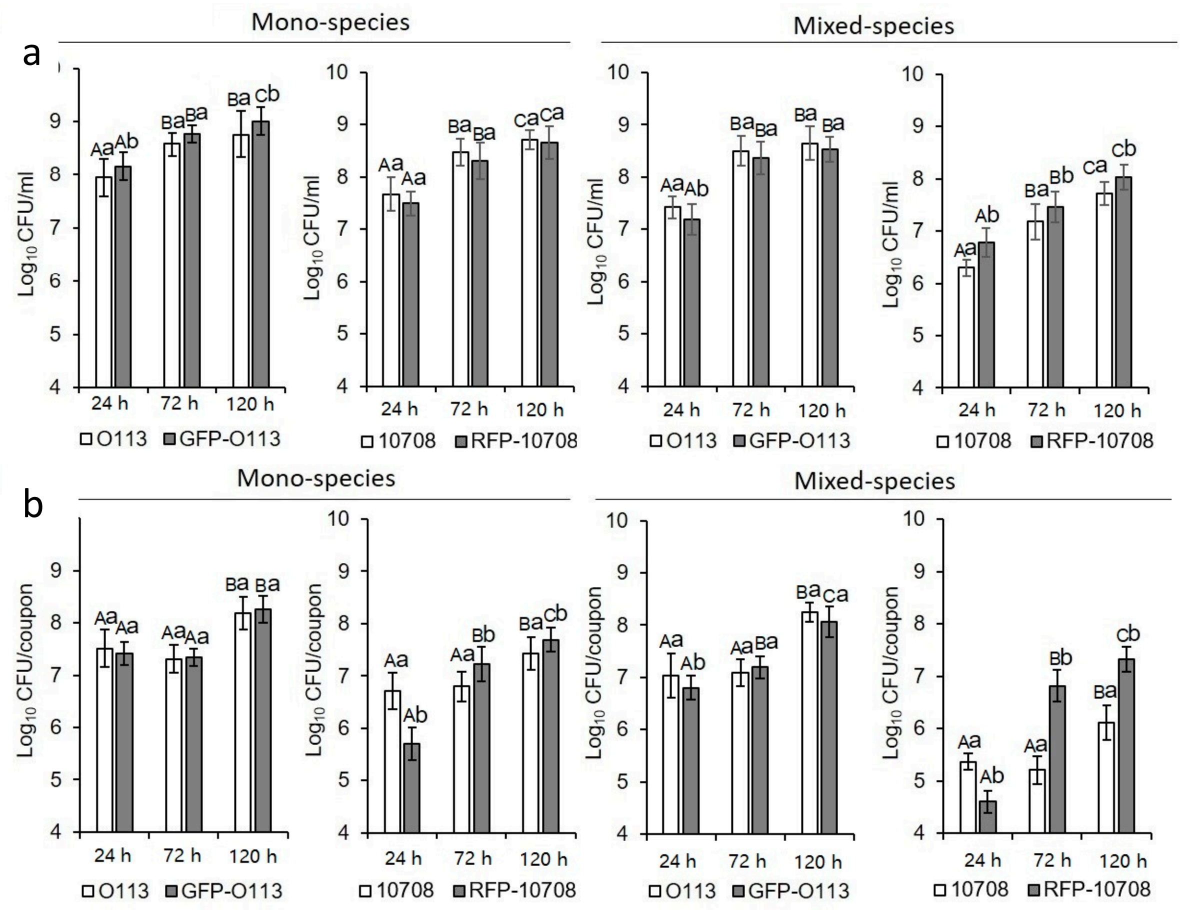

Figure S6. Cell enumeration of FP-labeled and parental *E. coli* O113 and *S.* Choleraesuis 10708 in their respective mono- and mixed-species planktonic cultures (a) and biofilms (b). The bars represent the mean values + standard deviations. Within each time, means with different lowercase letters differ ($P < 0.05$). Within each species, means with different uppercase letters differ ($P < 0.05$).
